# Supplementary material for: Comparative analysis of mRNA and protein degradation in prostate tissues indicates high stability of proteins
Source: Nat Commun. 2019 Jun 7;10:2524. doi: 10.1038/s41467-019-10513-5 (PMC6555818; doi:10.1038/s41467-019-10513-5)
Supplement: Supplementary file 1 — Supplementary Information [file 41467_2019_10513_MOESM1_ESM.docx]

**Supplementary Information**

**for**

**Comparative analysis of mRNA and protein degradation in prostate tissues indicates high stability of proteins**

Wenguang Shao ^1,#^, Tiannan Guo ^1,2,3,#,^*, Nora C Toussaint ^4,5^, Peng Xue ^1,6^, Ulrich Wagner ^7^, Li Li ^8^, Konstantina Charmpi ^8^, Yi Zhu ^1,2,3^, Jianmin Wu ^9^, Marija Buljan ^1^, Rui Sun ^2,3^, Dorothea Rutishauser ^7^, Thomas Hermanns ^10^, Christian Daniel Fankhauser ^10^, Cedric Poyet ^10^, Jelena Ljubicic ^7^, Niels Rupp ^7^, Jan H. Rüschoff ^7^, Qing Zhong ^7,11^, Andreas Beyer ^8^, Jiafu Ji ^12^, Ben C. Collins ^1^, Yansheng Liu ^13^, Gunnar Rätsch ^5,14^, Peter J. Wild ^7,15,^*, Ruedi Aebersold ^1,16,^*

1, Department of Biology, Institute of Molecular Systems Biology, ETH Zurich, Zurich, Switzerland

2, School of Life Sciences, Westlake University, 18 Shilongshan Road, Hangzhou 310024, Zhejiang Province, China

3, Institute of Basic Medical Sciences, Westlake Institute for Advanced Study, 18 Shilongshan Road, Hangzhou 310024, Zhejiang Province, China

4, NEXUS Personalized Health Technologies, ETH Zurich, Zurich, Switzerland

5, SIB Swiss Institute of Bioinformatics, Zurich, Switzerland

6, Institute of Biophysics, Chinese Academy of Sciences, Beijing, China

7, Institute of Surgical Pathology, University Hospital Zurich, Zurich, Switzerland

8, CECAD, University of Cologne, Cologne, Germany

9, Key laboratory of Carcinogenesis and Translational Research, Center for Cancer Bioinformatics, Peking University Cancer Hospital & Institute, Beijing, China

10, Department of Urology, University of Zurich, University Hospital Zurich, Zurich, Switzerland

11, Cancer Data Science Group, Children's Medical Research Institute, University of Sydney, Sydney, New South Wales, Australia

12, Key laboratory of Carcinogenesis and Translational Research, Department of Gastrointestinal Surgery, Peking University Cancer Hospital & Institute, Beijing, China

13, Department of Pharmacology, Cancer Biology Institute, Yale University School of Medicine, West Haven, USA

14, Department of Computer Science, ETH Zurich, Zurich, Switzerland

15, Dr. Senckenberg Institute of Pathology, University Hospital Frankfurt, Frankfurt am Main, Germany

16, Faculty of Science, University of Zurich, Zurich, Switzerland

#, These authors contributed equally

*, Corresponding authors: [aebersold@imsb.biol.ethz.ch](mailto:aebersold@imsb.biol.ethz.ch), [peter.wild@kgu.de](mailto:peter.wild@kgu.de), guotiannan@westlake.edu.cn

**Content**

**I. Supplementary Note 1** ----------------------------------------------------------------------------Page 4

**II. Supplementary Note 2** ---------------------------------------------------------------------------Page 9

**III. Supplementary Note 3** -------------------------------------------------------------------------Page 14

**IV. Supplementary Figures**------------------------------------------------------------------------Page 17

**V. Supplementary Tables**--------------------------------------------------------------------------Page 30

**VI. Supplementary References**--------------------------------------------------------------------Page 35

**Supplementary Note 1**

**A benchmarking study to validate the PIN algorithm**

To benchmark the PIN algorithm with an orthogonal method, we generated a set of “ground truth” samples, in which the levels of proteome degradation were known and independently validated. To generate this set of benchmarking samples, protein extracts of HeLa Kyoto cells were treated with the low specificity protease Proteinase K at different protease concentrations. Specifically, six samples of protein extracts (see A-F in **Table 1**, main text) were treated with sequentially increasing amounts of Proteinase K ranging from 0.0005 μg/μl to 0.02 μg/μl, while maintaining a constant substrate concentration of 1 μg/μl. All treated samples were incubated at 25℃ for five minutes and were rapidly transferred to heat at 100℃ for five minutes. To best simulate the practical situation of a clinical sample cohort and to set up the null distribution for outlier detection in the follow-up statistical test, nine samples were prepared as controls without treatment of Proteinase K. All samples were then subjected to complete tryptic digestion prior to mass spectrometric analysis. Each sample, including the controls, were prepared in two biological replicates, and were then acquired in the same way as described in the main text on a TripleTOF 5600 mass spectrometer operated in SWATH-MS mode. Two technical replicates were measured per sample (see **Table 1**, main text). In addition, 28 shotgun MS measurements, two biological replicates and two technical replicates from seven samples (six treated samples plus one control sample), were acquired for SWATH assay library construction. In total, the benchmarking dataset consists of 60 SWATH-MS injections and a SWATH assay library constructed from 28 shotgun injections.

As an orthogonal validation method, sodium dodecyl sulfate polyacrylamide gel electrophoresis (SDS-PAGE) was applied to the biological replicates of six treated samples and one control sample. As shown in **Fig. 2a** (main text), the treated samples demonstrated strong and progressive evidence of proteome degradation as expected – intact proteins breaking into their smaller sub-units, resulting in the presence of a larger number of low molecular weight bands in the gel image and concurrent depletion of the higher molecular weight bands. These indicators of protein degradation positively correlate with the amount of protease added. These samples, therefore, constitute a valuable resource to benchmark the mass spectrometric PIN score.

To analyze the 60 SWATH-MS injections generated from the benchmarking samples, we used the computational workflow described in the main text with several modifications. OpenSWATH was used with the same set of parameters described in the main text, except the modifications for *m/z* and RT windows. The SWATH assay library built from 28 shotgun injections of the benchmarking samples contained fragment ions of 29827 tryptic peptides and 10289 semi-tryptic peptides. Using the same set of parameters described in the main text, we ran PyProphet to estimate the q-value to facilitate FDR control, and then ran TRIC to perform the feature alignment to re-rank peak groups. As a result, we obtained fragment ion peak groups for 17020 proteotyptic peptide ions (12735 fully tryptic and 4285 semi-tryptic) representing 2232 proteins. The result of these analyses was a quantitative peptide matrix for the benchmarking sample set.

We then calculated the PIN score as an indicator of proteome integrity of each sample in the benchmarking datasets, using the peptide quantitative matrix. All replicates were merged by taking mean values of intensities. Not surprisingly, the PIN values of the treated samples decreased with progressing protein degradation. Interestingly, the PIN values showed a strong linear relationship (R^2^: 0.94) with the amount (on a logarithmic scale) of Proteinase K added, suggesting that PIN values are accurate indicators of the degree of protein degradation (**Supplementary Fig. 2**). After statistical analysis that estimates a *P* value indicating the probability of observing such a specific PIN value under the null hypothesis that the sample was not degraded (see Method in main text), we were able to confidently identify the samples with protease treatment (**Table 1** and **Fig. 2b**, main text) with different statistical significances. For example, three samples treated with the highest amount of protease added were identified as most extensively degraded, with *P* values below 1e-5; next, two samples were identified as degraded with *P* values between 1e-5 and 0.001; the sample treated with the minimal amount of protease was identified as intermediately degraded with a *P* value of 0.04 (**Fig. 2b**, main text). Overall, we showed that the PIN workflow provides precise measurements of proteomics samples to infer protein degradation, and is sensitive and robust to detect degraded samples with various extents.

Interestingly, in the benchmarking dataset, we identified 105 semi-tryptic peptide ions from 79 proteins, from which fully tryptic peptides were not detected at all. This means in the benchmarking study, these 79 proteins could be detected only by semi-tryptic peptides. Thus, we provide these 105 semi-tryptic peptide ions in the format of spectral library, to facilitate future targeted DIA/SWATH analysis for the community. The library is publicly available on https://github.com/ProteomicsTools/PIN/tree/master/data.

To further validate the PIN algorithm, we compared the PIN results with results obtained from a published degradation scoring scheme that is based on the degradation of a single protein [1]. The authors used the number of semi-tryptic peptides and the formation of “ladder” peptides (*i.e.* peptides sequentially cleaved by the protease) from fibrinogen alpha to monitor protein degradation in blood samples [1]. We used the SWATH-MS data of 68 prostate tissue samples (**Fig. 3b** in the main text) to benchmark the PIN score against the formation of ladder peptides. We detected fragment ion signals for 426 ladder peptides from 26 proteins, suggesting the presence of protein degradation in this set of clinical tissues. The number of ladder peptides detected in each sample showed a negative correlation (Pearson r: -0.494; *P* value: 0.03) with the PIN, demonstrating the effectiveness of our PIN scoring system (**Supplementary Fig. 3**). Instead of relying on the degradation state of a single protein, *e.g.* fibrinogen alpha as applied in the published literature [1], our PIN score uses results of large-scale protein measurements to indicate the level of proteome integrity. Moreover, the PIN workflow is based on SWATH-MS, an unbiased and accurate proteomic technique operating in a highly reproducible and quantitatively accurate manner, allowing an accurate estimation for PIN values based on MS/MS intensities rather than the peptide/spectral counting of “ladder” peptides.

In conclusion, in this benchmarking study, we generated a set of calibrated samples with various degrees of protein degradation by introducing different amounts of protease, and further visualized and validated the level of protein degradation independently by SDS-PAGE. By applying our PIN workflow to the SWATH-MS measurements of the same set of the samples as well as benchmarking against a scoring scheme based on a single protein as proposed in a previous study, we show that the PIN algorithm provides precise measurements (*i.e.* PIN values) to indicate various degrees of protein degradation on the sample level, and the workflow overall is sensitive and robust to identify degraded samples of various levels with correct statistical confidences.

**Supplementary Note 2**

**General utility of the PIN algorithm**

To demonstrate the general utility of the PIN algorithm, we applied it to several additional and independent datasets of clinical proteomics research studies [2-5] that included various types of clinical samples (specifically prostate tissue; breast tissue; gastric tissue and human plasma), different sample storage methods (specifically formalin-fixed paraffin-embedded (FFPE) and fresh frozen (FF)), different MS instruments, data acquisition and proteomics techniques (specifically Data dependent acquisition (DDA) vs. Data independent acquisition (DIA); iTRAQ labeling vs. label-free quantification; whole samples vs. fractionated samples). Pertinent details of these studies were summarized in **Supplementary** **Table 4**. The diversity of these studies allowed us to demonstrate the general utility of the PIN algorithm and to investigate generic issues related to protein degradation in clinical samples.

In total, we used the PIN algorithm, along with several widely used computational proteomics workflows (e.g. MaxQuant; OpenSWATH), to (re-)process and analyze results from **1546 MS injections** measured from **522 clinical samples**. The detailed results of the individual studies were summarized in the following.

*Study 1: CPTAC breast cancer tissue samples*

We applied the PIN algorithm to 900 iTRAQ Thermo Q Exactive measurements (iTRAQ 4X; high pH reversed-phase fractionations) of 108 TCGA breast cancer samples [2]. The original report stated that 28 samples showed extensive degradation at the protein level, concluded by additional analyses [2]. These analyses included manual investigation of protein distributions of abundance, absence and iTRAQ ratios, and performing degradation-related gene set enrichment analysis.

We re-searched the whole set of 900 MS measurements using MaxQuant with the same parameters described in the original report [2], except that the enzyme type was set to semi-tryptic, thus allowing the detection of semi-tryptic peptides as potential products of protein degradation. By applying the PIN algorithm to thus obtained search results, we identified 24 samples (22.2%), out of 108 samples, as significantly (*P* value < 0.01) degraded (**Supplementary Fig. 6**). The 24 samples identified as substantially degraded based by the PIN score showed a high degree (22 samples; 91.7%) of overlap with the samples identified as degraded in the original report (**Supplementary Fig. 7**). Among the six samples that had been identified as degraded in the original report but not by the PIN score, two showed PIN based *P* values around 0.02 and two showed PIN based *P* values around 0.05, indicating that these samples in fact showed intermediate levels of proteome degradation.

*Study 2: Gastric* *cancer tissue samples*

We applied the PIN algorithm to 40 SWATH-MS measurements of 18 fresh frozen gastric cancer tissue samples [3]. Of these 18 samples, 11 samples demonstrated extensive RNA degradation (RIN < 1.8).

PIN analysis identified 3 of the 18 samples as significantly (*P* value < 0.01) degraded at the protein level (**Supplementary Fig. 8**). Interestingly, as was the case with the prostate tissue samples (in the main text), the samples showing degraded proteins neither overlapped exclusively with RNA extensively degraded samples, nor samples showing insignificant RNA degradation (**Supplementary Fig. 9**). Furthermore, the PIN values showed no significant (*P* value: 0.8796, calculated by Welch’s *t*-test) differences between samples with degraded or non-degraded RNA (**Supplementary Fig. 9**), suggesting negligible dependency between transcriptome and proteome degradation in this set of gastric samples. Our data indicate that the PIN algorithm is useful to detect differences in protein integrity of the gastric tissue samples, even though in this sample set the difference was small. The available results did not allow us to determine whether the detected differences in protein degradation state was due to technical issues or biological reasons (e.g. endogenous proteolysis in the stomach). The data also confirmed the observation in the main text that the pre-analytical variables causing the respective mRNA and protein degradation are decoupled.

*Study 3: Prostate* *tissue samples*

We applied the PIN algorithm to 224 SWATH-MS measurements of 96 prostate tissue samples, consisting of 48 formalin-fixed paraffin-embedded (FFPE) samples and 48 counterpart fresh frozen (FF) samples [4] that had been stored in the respective state for four to eight years [4].

PIN analysis identified 6 samples (6.25%), out of 96 samples as significantly (*P* value < 0.01) degraded at the protein level (**Supplementary Fig. 10**). Further, compared with fresh frozen samples, FFPE samples showed a significantly (*P* value: 1.038e-12, calculated by Welch’s *t*-test) higher level of protein stability, as measured by the PIN values (**Supplementary Fig. 11**). Remarkably, all six samples with significantly degraded proteomes were fresh frozen, suggesting that FFPE effectively prevents protein degradation in clinical samples, possibly due to protease inactivation after formalin fixation. These results indicate that the PIN algorithm is applicable to clinical samples stored under the two most commonly used protocols, fresh frozen and FFPE. The data also clearly show that FFPE is the preferred sample storage method for longer term storage, because it reduces the effects of inevitable protein degradation in clinical settings.

*Study 4: Human blood plasma*

We applied the PIN algorithm to a longitudinal twin study consisting of 232 plasma samples from 116 patients, who donated their blood plasma twice within a time span of 2-7 years [5]. The original study was designed to assess the quantitative variability of plasma proteins and to estimate the relative contribution of heritability and environment to this observed variability. The previously acquired SWATH-MS dataset of this large sample cohort, together with the associated spectral library that had already contained ions of semi-tryptic peptides [5, 6], was re-visited and directly analyzed by PIN.

As shown in **Supplementary Fig. 12**, there was no significantly (*P* value < 0.01) degraded plasma sample in the Liu, Y., *et al.* study. This observation further confirmed the results obtained in a previous investigation focusing on plasma proteome stability [1]. Zimmerman et al. reported the remarkable stability of plasma proteome, even after storage for a week at 4 ℃ or at room temperature in EDTA plasma tubes [1].

**Supplementary Note 3**

**Manual of the PIN algorithm**

*Software description*

This document describes a computational method, namely PIN (proteome integrity number), to assess a quantitative measure of protein stability directly from bottom-up proteomic datasets.

By quantifying the relative abundance of semi-tryptic peptides (i.e. the likely products of protein degradation), an individual protein integrity score (iPIS) was first calculated for each measured protein. A per-sample PIN was then derived to summarize a proteome-wide measurement, indicating the level of protein degradation.

This method has been applied to several clinical cohorts of prostate tissue samples, breast cancer tissue samples, gastric cancer samples and human plasma samples, respectively in which degradation is sometimes inevitable during the sample preparation. The datasets analyzed also represent the types of most commonly used clinical specimens, fresh frozen tissue, FFPE tissue and plasma and the most common mass spectrometric acquisition methods. Overall, the data indicate that the PIN algorithm is broadly applicable for the assessment for the proteome integrity in clinical studies.

*Installations*

--- Installation of development version from GitHub

*$ library(devtools)*

*$ install_github("shaowenguang/PIN")*

--- Installation of released version from CRAN

*$ install.packages("PIN")*

*Step-by-step procedures*

Step 1: to load the package.

*$ library(PIN)*

Step 2: to import necessary files (search results, spectral library and sample annotation table). It will return a peptide table in long format.

*$ peptides <- generate_peptide_table(search_results="./openswath_search_results.tsv", sample_annotation="./sample_annotation_table", sptxt="./spectral_library.sptxt", remove_prefixInFileName = FALSE, normalize_intensity = TRUE)*

Available parameters:

| **Parameter** | **Options** | **Default** | **Explanation** |
| --- | --- | --- | --- |
| remove_prefixInFileName | True or False | False | to automatically detect and remove common prefix in file names. |
| normalize_intensity | True or False | True | to remove potential batch effects by equalizing median intensities of each individual mass spectrometric injections. It is simple and effective, as one of common ways to normalize proteomics data |

Step 3: to perform PIN analysis.

*$ perform_PIN_analysis(peptides)*

Available parameters:

| **Parameter** | **Options** | **Default** | **Explanation** |
| --- | --- | --- | --- |
| intensity_scale | Raw, Sqrt and Log | Sqrt | different scales of intensities : Raw intensities; square root of intensities and log-transformed intensities. |
| remove_zero_rows | True or False | FALSE | to remove proteins detected ONLY by semi-tryptic peptides. |

*Outputs*

| **File Name** | **Description** |
| --- | --- |
| PIN.tsv | PIN values and their associated *P* values, for each sample |
| iPIS.tsv | individual Protein Integrity Score, for each individual proteins |
| lib_peptide.tsv | a temp file that records the assay library information |

*Examples*

See **Supplementary Fig. 13**.

**Supplementary Figures**


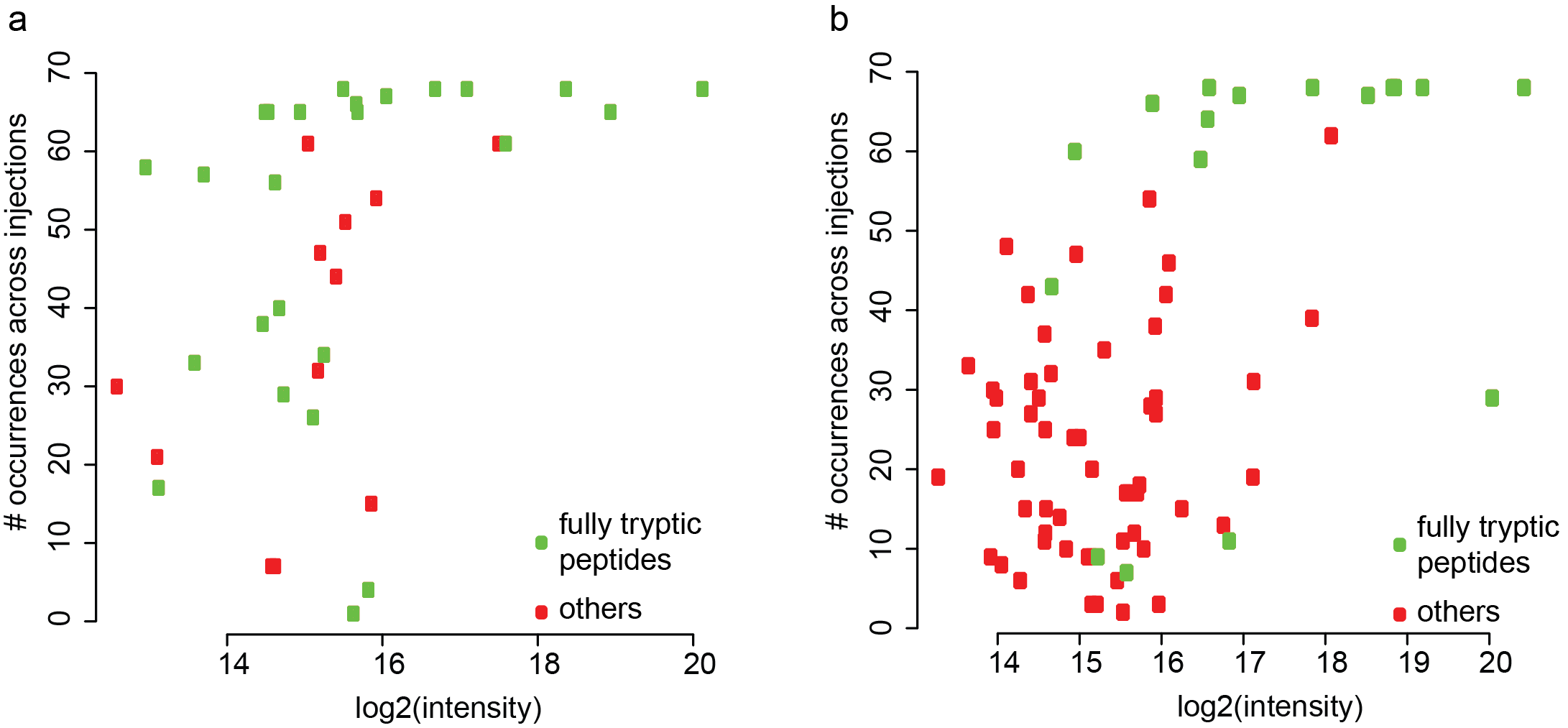


**Supplementary Figure 1**

Two representative proteins, (**a**) P20774|MIME_HUMAN and (**b**) P07288|KLK3_HUMAN, illustrating different behaviors of fully tryptic peptides (colored in green) and semi-tryptic peptides (colored in red), based on their averaged intensities and the occurrences across the sample cohort studied.


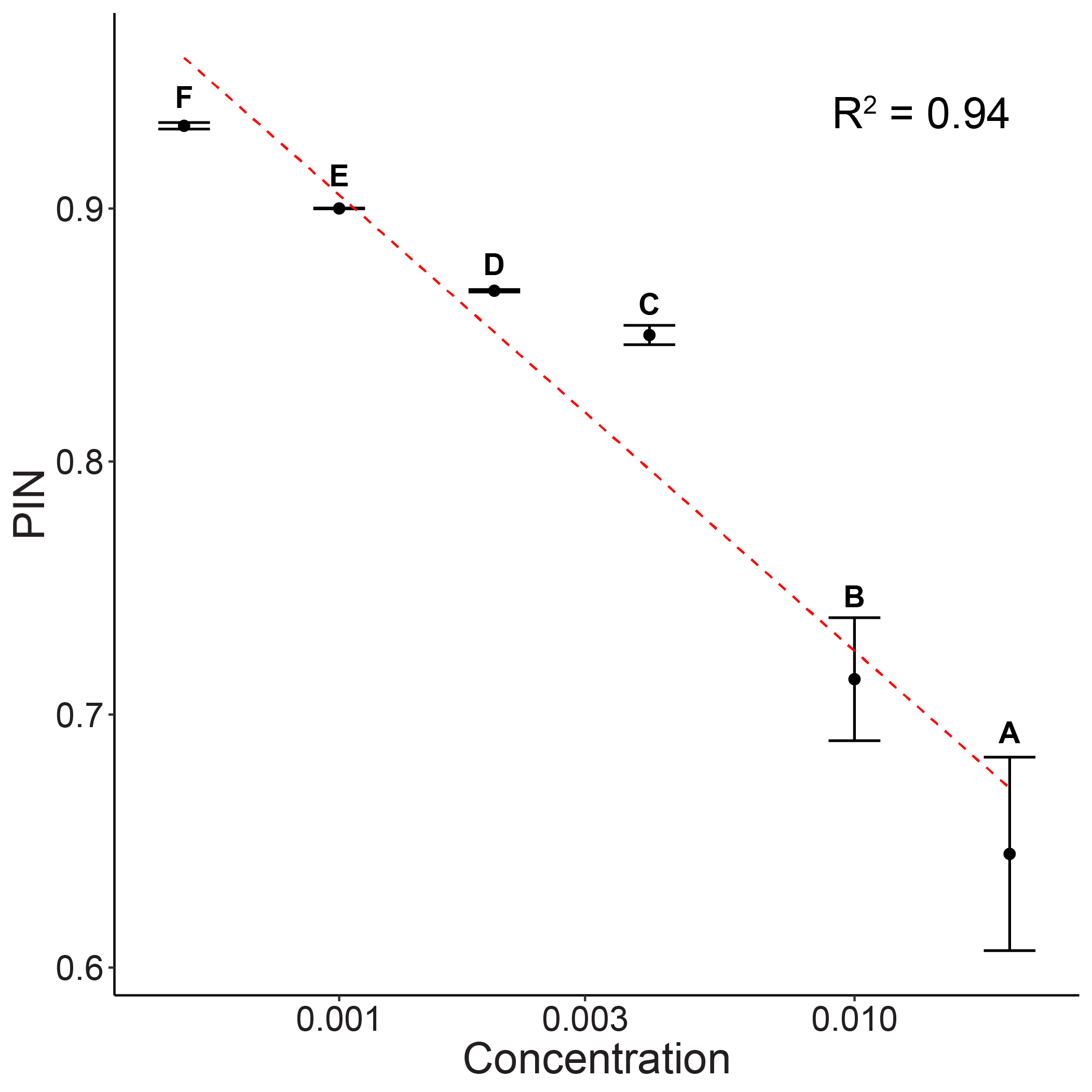


**Supplementary Figure 2**

Scatter plot of the concentrations (on a logarithmic scale) and resulting PIN values of the benchmarking samples defined in Supplementary Table 1. The dashed line in red was fitted by linear regression and the linear correlation had an R^2^ of 0.94. Data are represented as mean ± s.d. (n=4).


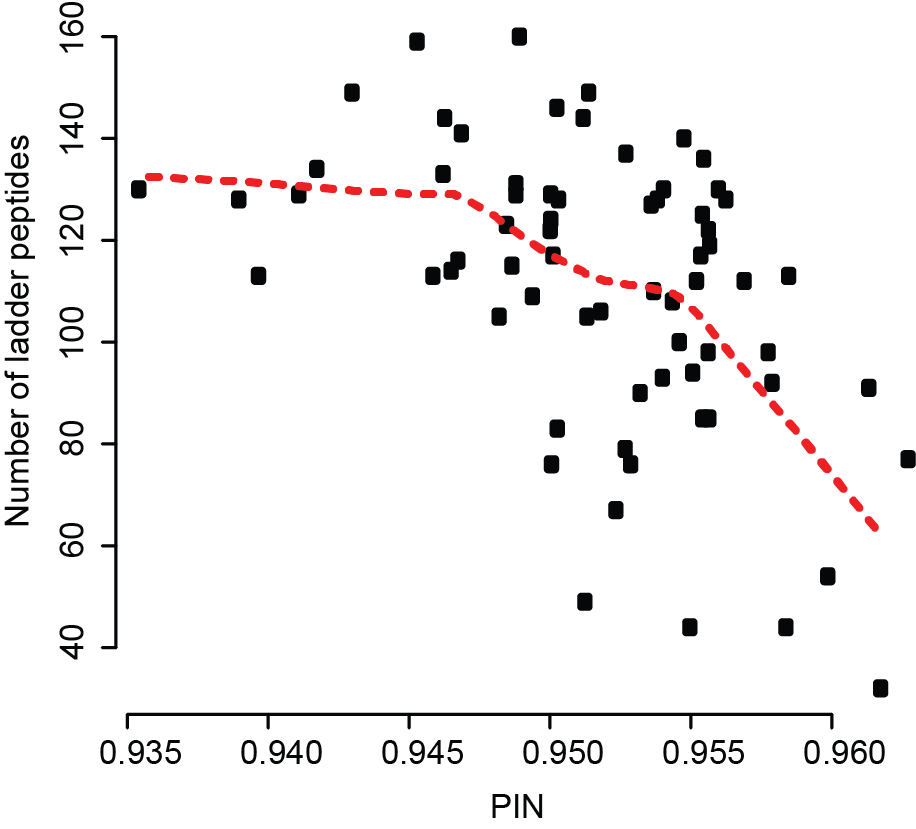


**Supplementary Figure 3**

Scatter plot demonstrating a negative correlation between PIN values and the number of ladder peptides according to [1]. The red curve was fitted by LOWESS (locally weighted scatterplot smoothing).


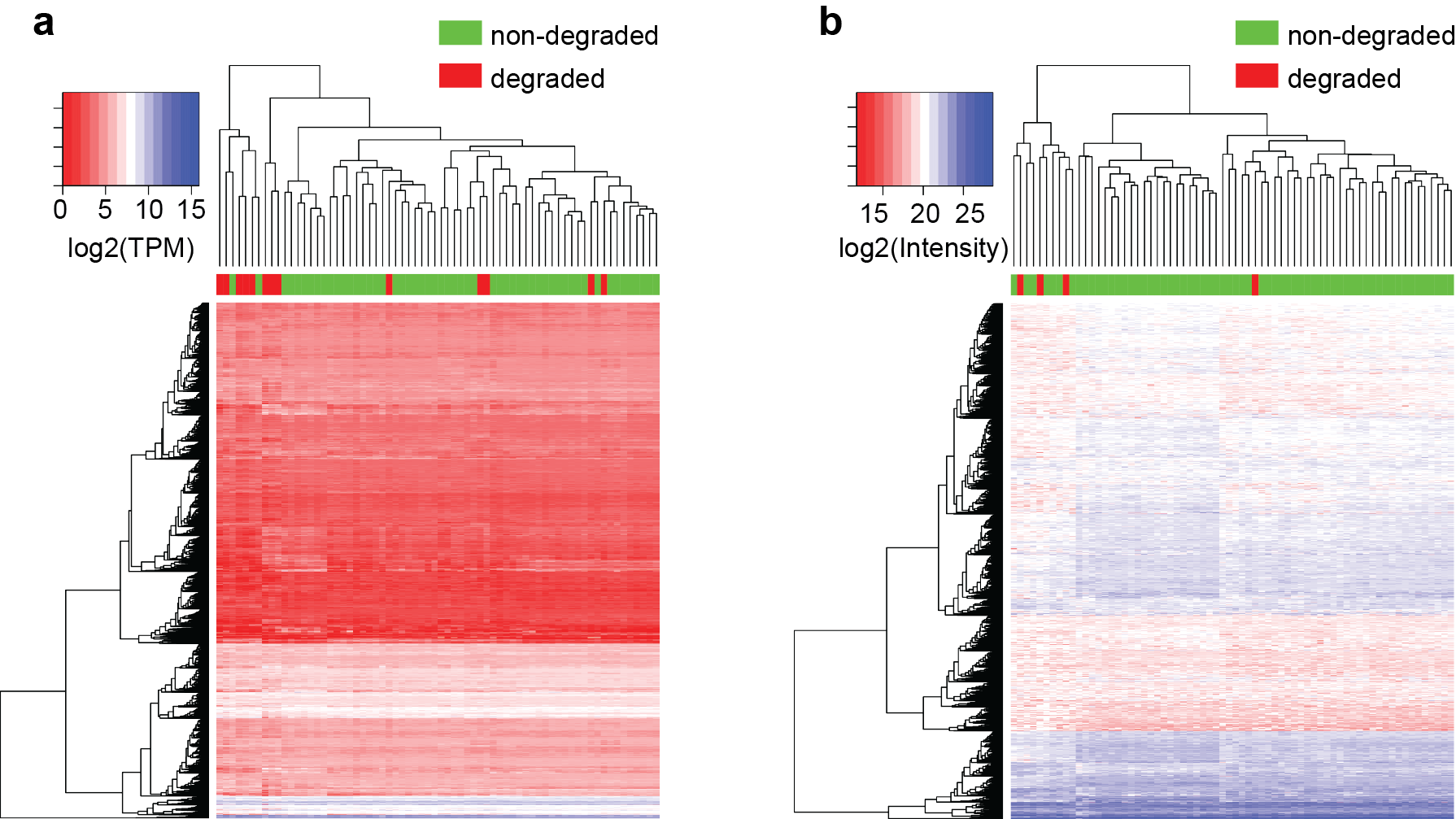


**Supplementary Figure 4**

Impact of degradation on global mRNA expression profile **(a)** and protein expression profile **(b).** The mRNA expression profile was generated using log2 transformed transcripts per million (TPM) values, from genes whose averaged TPM values were larger than 4. Protein abundance was estimated by summing the top 6 most intense fragment ion peak areas from the most intense peptide ion. Normalization was performed by equalizing medians of each injection at the peptide ion level and protein abundance was then log2 transformed. The same algorithm, heatmap.2 function from the R package gplots, was used to perform the hierarchical clustering and plot the heatmaps of mRNA and protein expression based on log2 transformed TPM values and protein abundances, respectively.


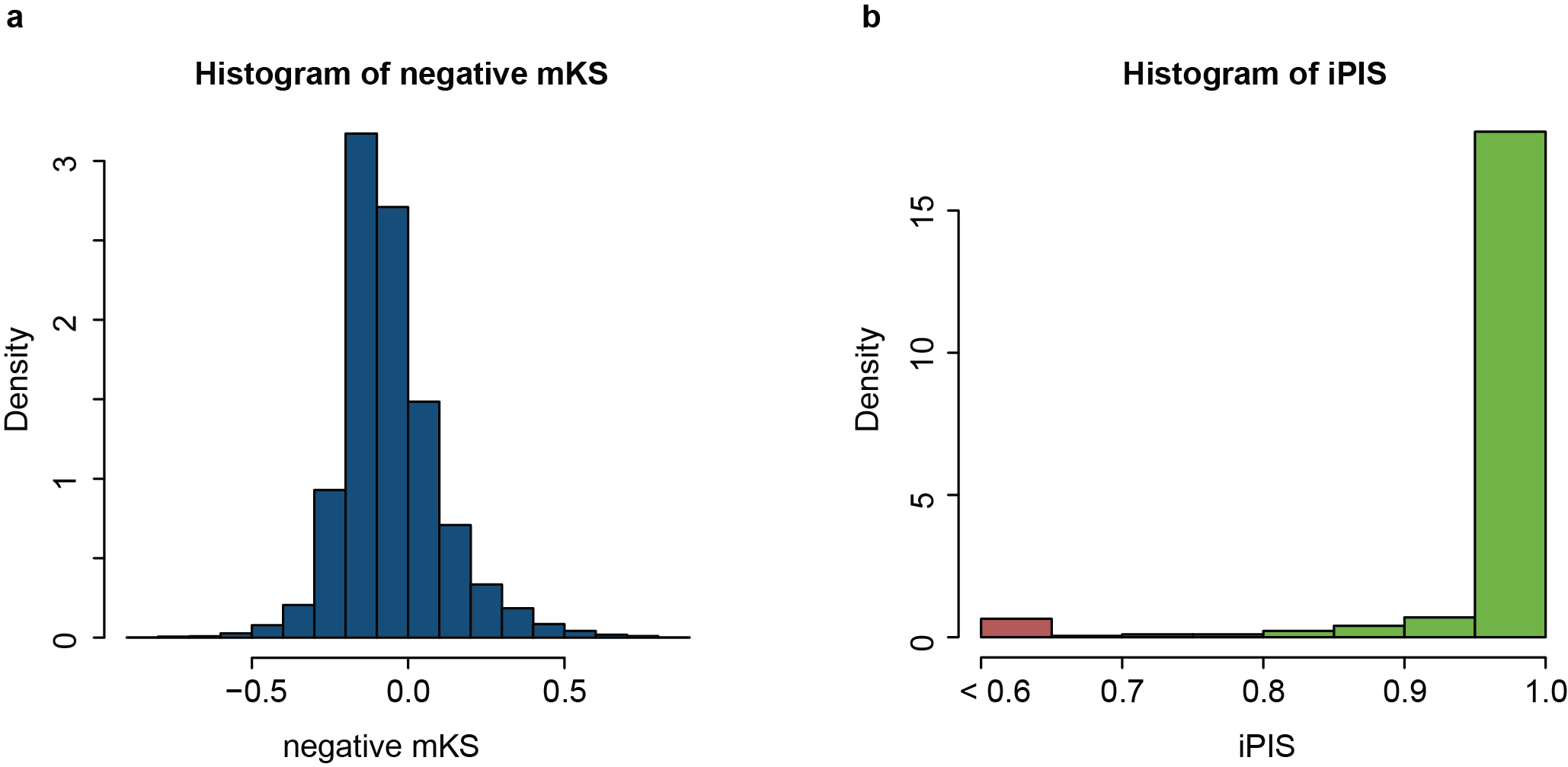


**Supplementary Figure 5**

Density plots of (**a**) negative mKS and (**b**) iPIS illustrating different degradation profiles of transcripts and proteins. Proteins with iPIS scores below 0.8, called degradation prone proteins, are marked in red.


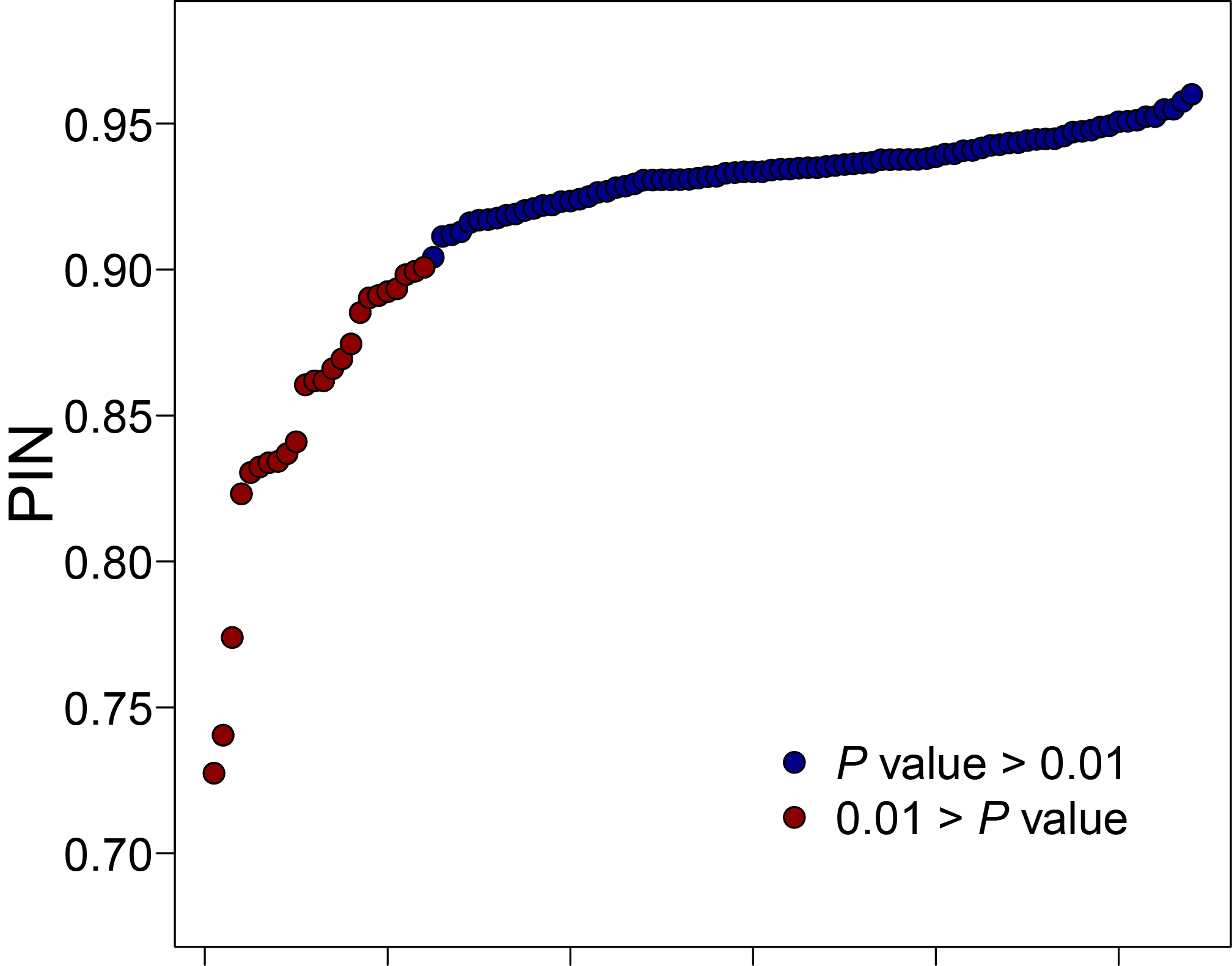


**Supplementary Figure 6**

Scatter plot of sorted PIN values of 108 breast tissue samples. 24 samples with significant (*P* value < 0.01) protein degradation are marked in dark red.


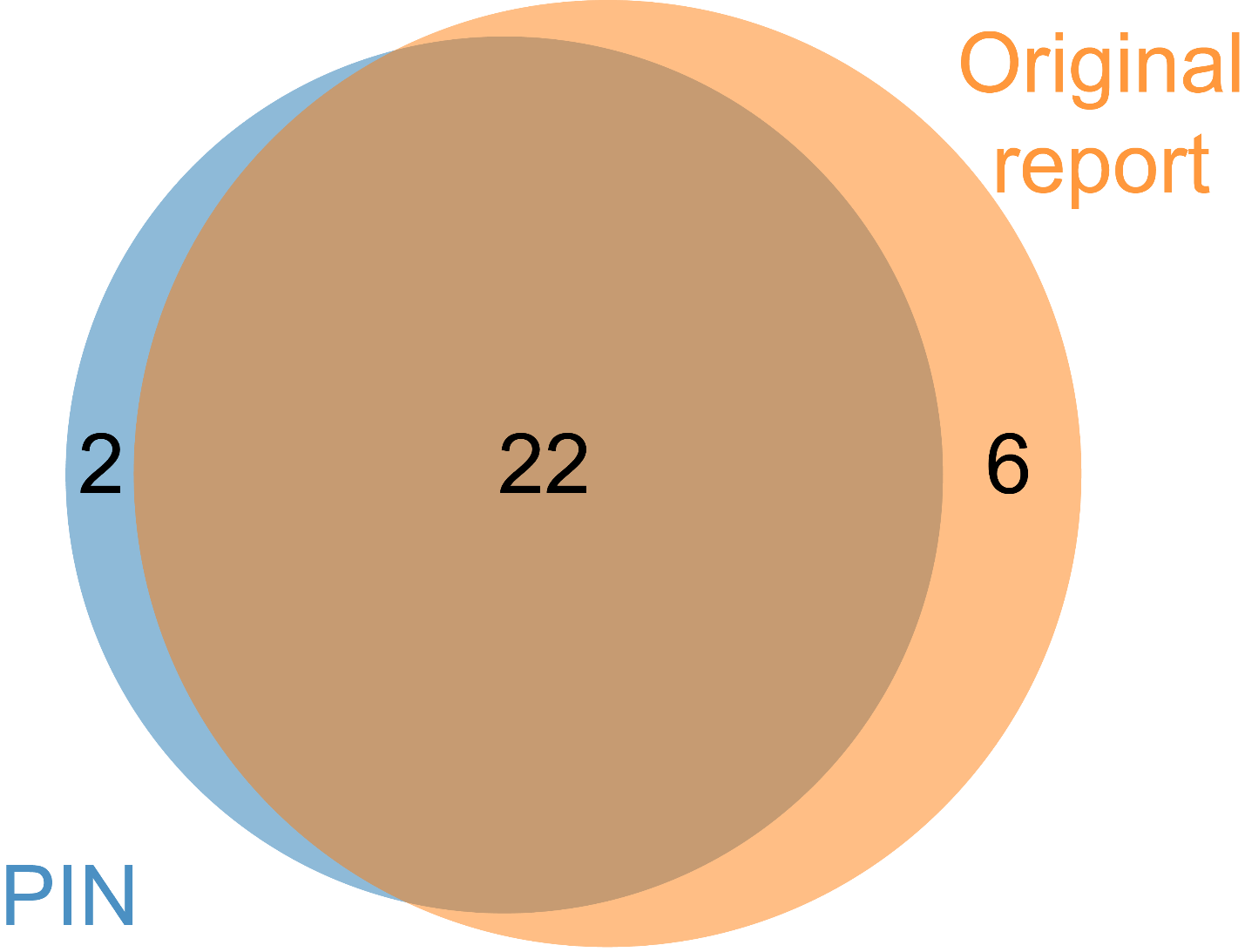


**Supplementary Figure 7**

Venn diagram of samples showing significant protein degradation as identified by the PIN score or reported in the original report [2].


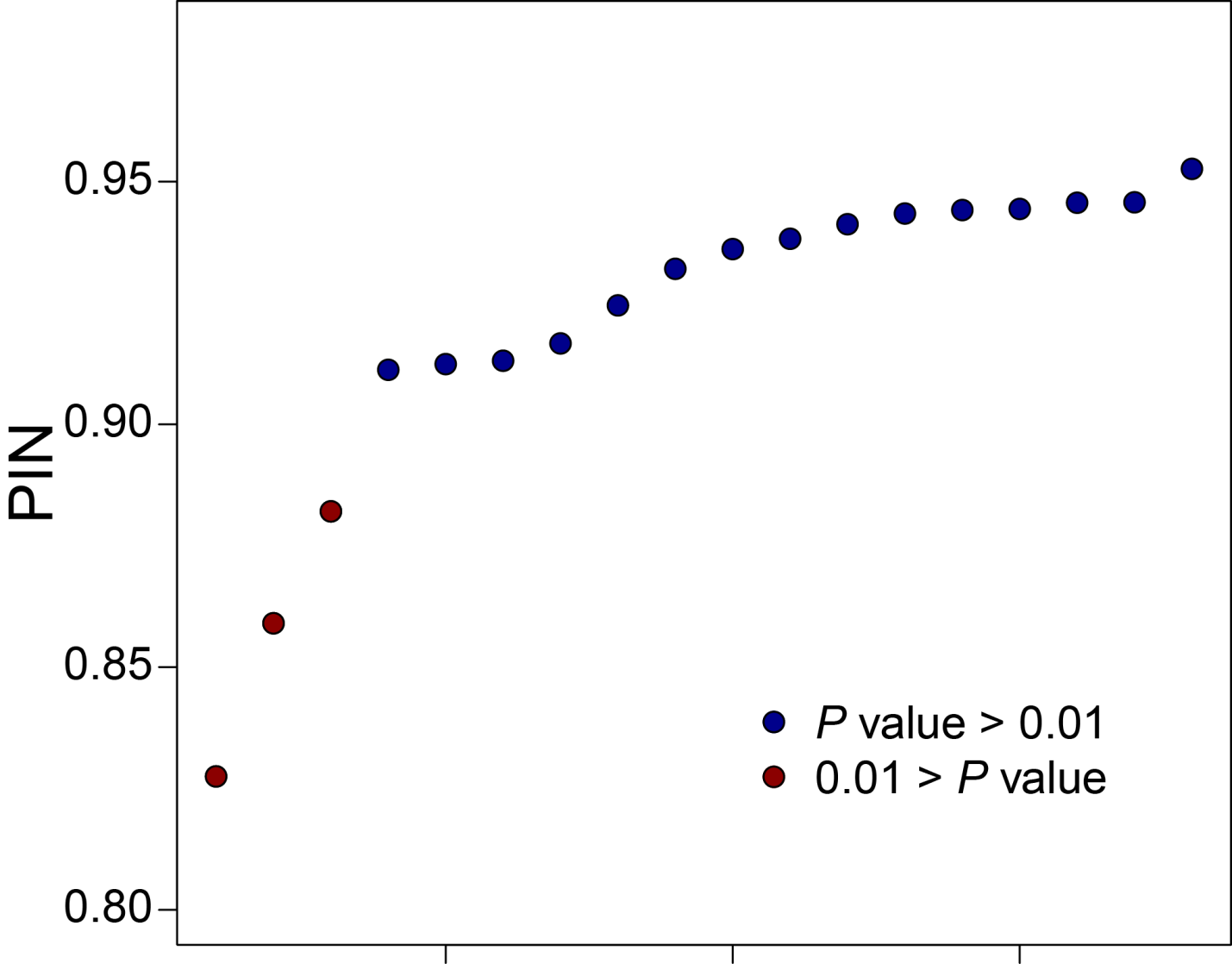


**Supplementary Figure 8**

Scatter plot of the sorted PIN values of 18 gastric cancer tissue samples. Three samples with significant (*P* value < 0.01) protein degradation are marked in dark red.


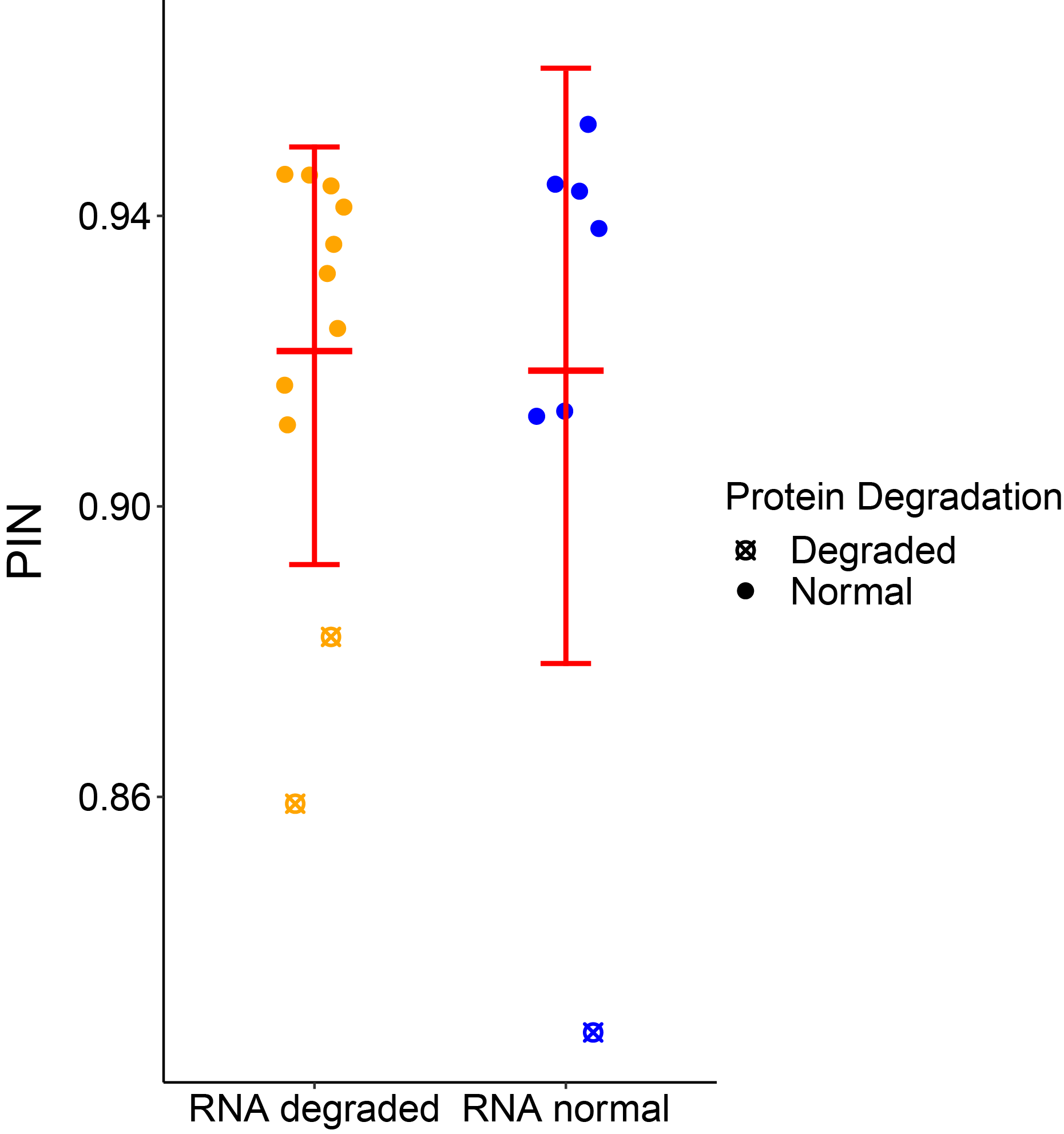


**Supplementary Figure 9**

Dot plot of the PIN values of 18 gastric tissue samples, grouped by the status of RNA degradation measured by RIN. Three samples with significant (*P* value < 0.01) protein degradation are labeled as circle with cross. Data are represented as mean ± s.d.; *n* = 11 for RNA degraded samples and *n* = 7 for RNA non-degraded samples.


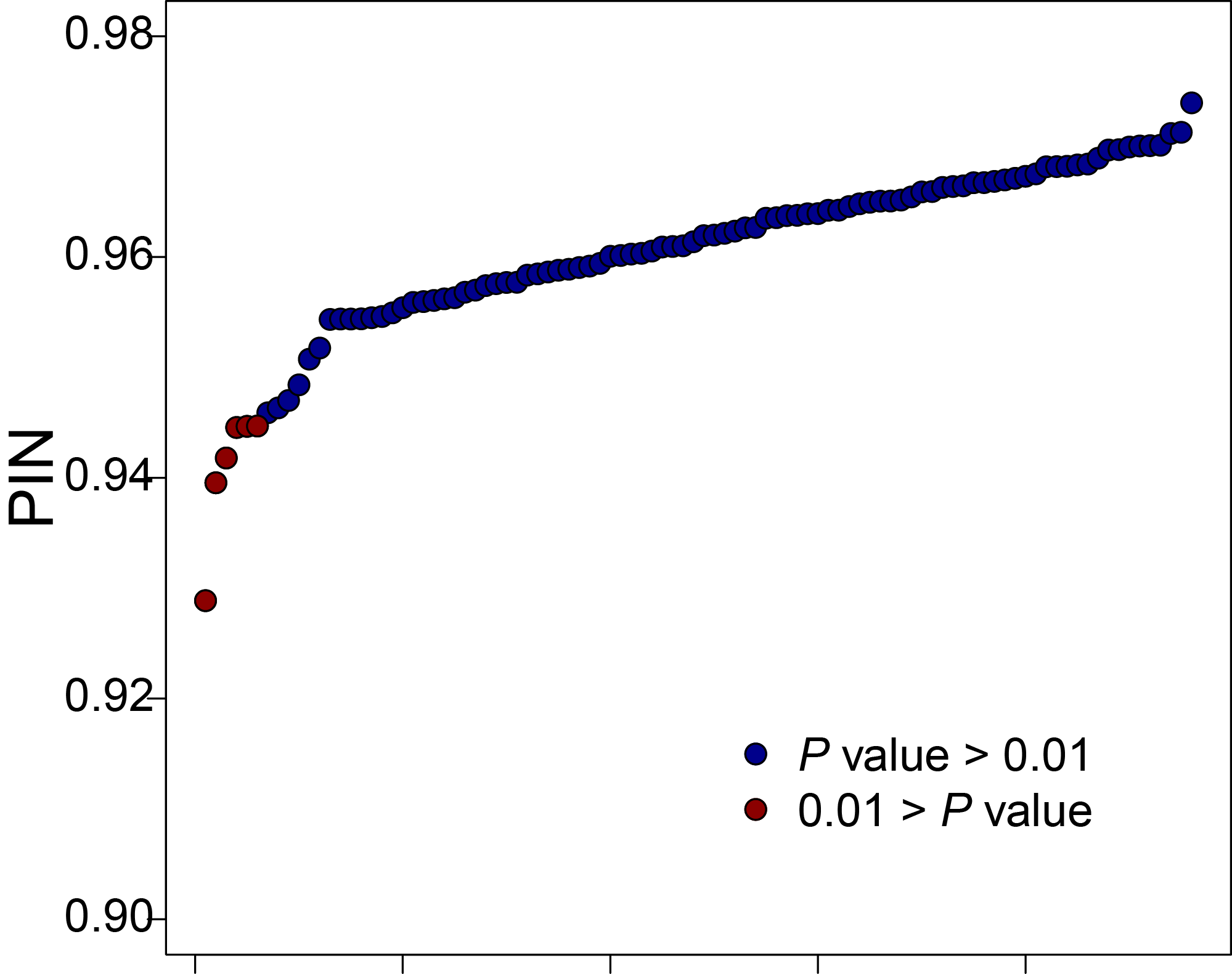


**Supplementary Figure 10**

Scatter plot of the sorted PIN values of 96 prostate tissue samples. Six samples with significant (*P* value < 0.01) protein degradation are marked in dark red.


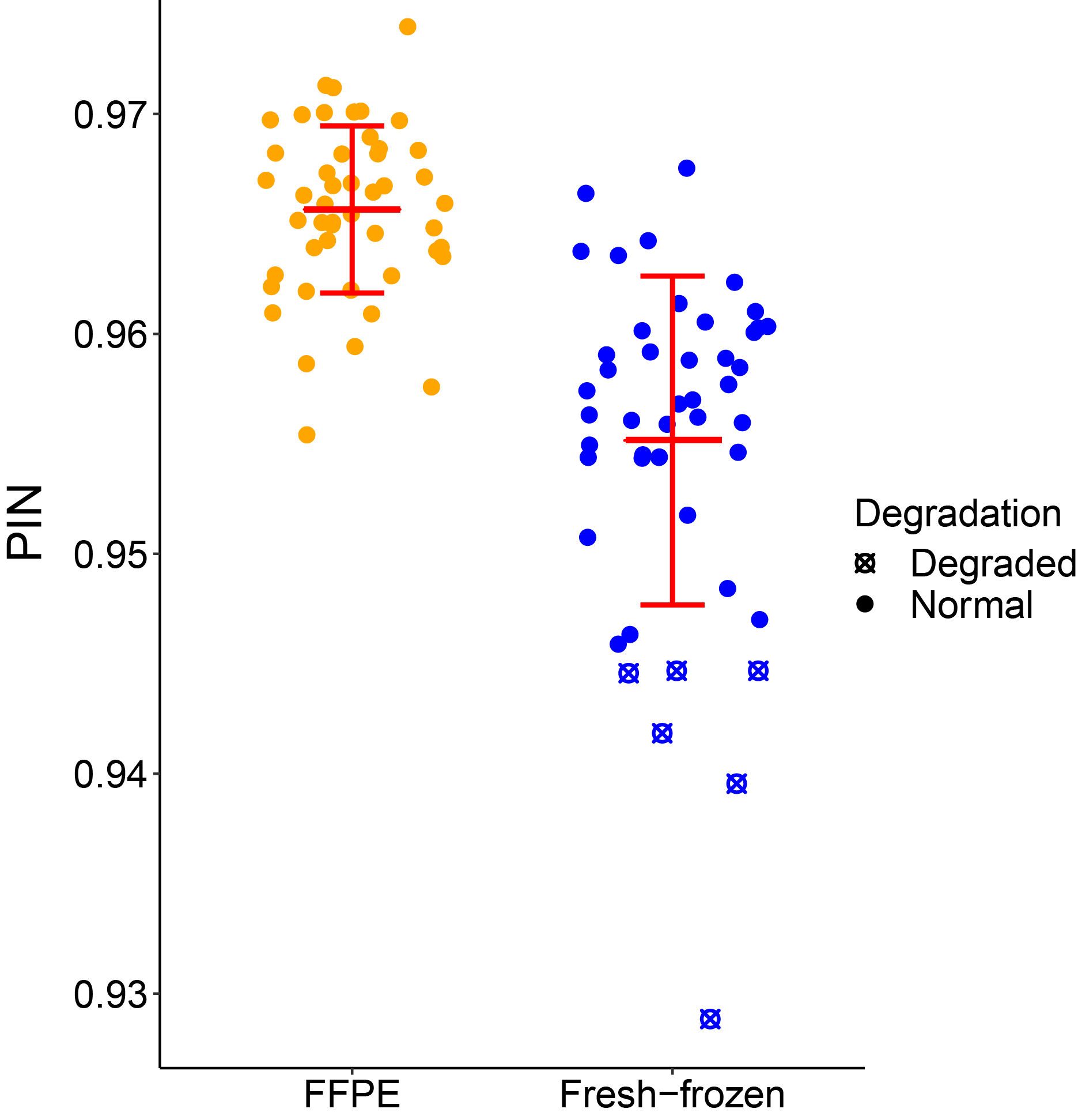


**Supplementary Figure 11**

Dot plot of the PIN values of 96 prostate tissue samples, grouped as FFPE or fresh frozen samples. The six samples with significant (*P* value < 0.01) protein degradation are labeled as circle with cross. Data are represented as mean ± s.d.; *n* = 48 for FFPE samples and *n* = 48 for fresh frozen samples.


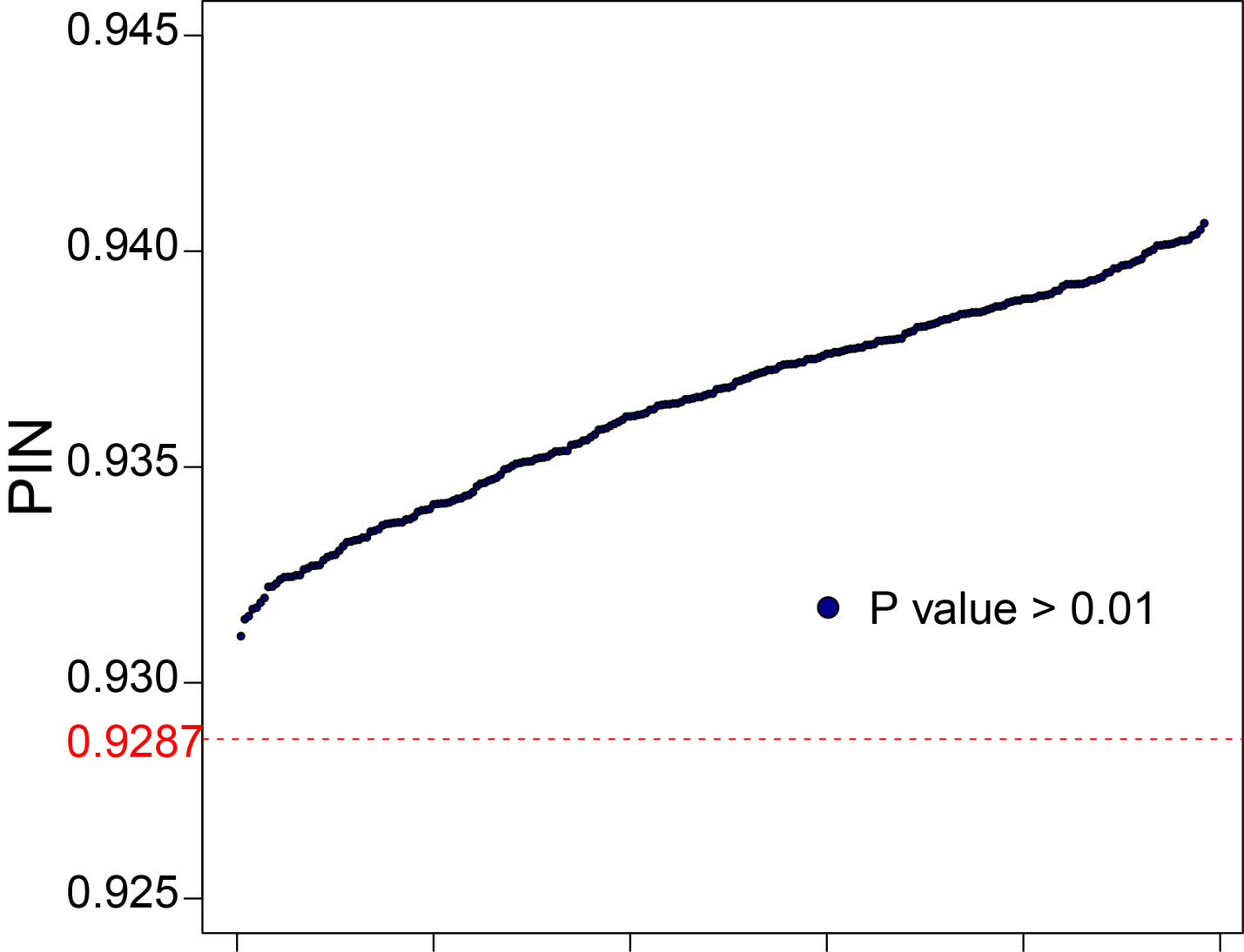


**Supplementary Figure 12**

Scatter plot of the sorted PIN values of 232 human plasma samples. None of the samples were identified with significantly degraded proteome. The horizontal dashed line in red (y=0.9287) indicates the estimated PIN score of significantly degraded proteome (*P* value 0.01).

**
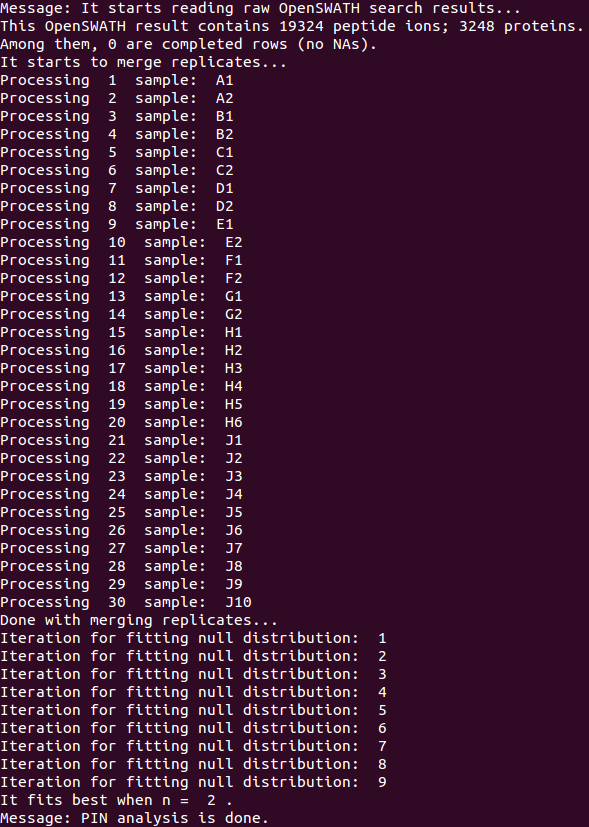
**

**Supplementary Figure 13**

The screenshot of running the benchmarking dataset in the Supplementary Note 1.

**Supplementary Tables**

**Supplementary Table 1 |** PIN values sorted in ascending order, with their associated *P* values of all the biological replicates measured in the benchmarking study.

| **Sample** | **Biological replicate** | **PIN** | ***P* value** |
| --- | --- | --- | --- |
| A | 2 | 0.6179 | 1.03E-20 |
| A | 1 | 0.6720 | 6.35E-17 |
| B | 1 | 0.6968 | 2.76E-15 |
| B | 2 | 0.7312 | 4.11E-13 |
| C | 1 | 0.8473 | 1.86E-06 |
| C | 2 | 0.8527 | 3.60E-06 |
| D | 1 | 0.8672 | 2.07E-05 |
| D | 2 | 0.8678 | 2.24E-05 |
| E | 2 | 0.8999 | 0.0010 |
| E | 1 | 0.9001 | 0.0010 |
| F | 1 | 0.9318 | 0.0356 |
| F | 2 | 0.9336 | 0.0435 |
| Control | 2 | 0.9573 | 0.4529 |
| Control | 1 | 0.9589 | 0.5128 |
| Control | 18 | 0.9619 | 0.6311 |
| Control | 11 | 0.9627 | 0.6596 |
| Control | 12 | 0.9628 | 0.6663 |
| Control | 16 | 0.9636 | 0.6947 |
| Control | 10 | 0.9639 | 0.7074 |
| Control | 17 | 0.9642 | 0.7198 |
| Control | 8 | 0.9643 | 0.7225 |
| Control | 15 | 0.9644 | 0.7261 |
| Control | 9 | 0.9648 | 0.7407 |
| Control | 14 | 0.9650 | 0.7483 |
| Control | 13 | 0.9650 | 0.7491 |
| Control | 6 | 0.9651 | 0.7538 |
| Control | 7 | 0.9651 | 0.7539 |
| Control | 5 | 0.9652 | 0.7557 |
| Control | 4 | 0.9652 | 0.7565 |
| Control | 3 | 0.9652 | 0.7573 |

**Supplementary Table 2** **|** Detailed information of the patients involved in the study.

| Patient ID | Gleason TA1 specific | Age of patient at operation | Blood PSA level at diagnosis (ng/ml) | ProCOC ID | Sample collection (Year) |
| --- | --- | --- | --- | --- | --- |
| 1 | 3+3 | 57 | 4.4 | 12 | 2008 |
| 2 | 4+3 | 54 | 19.9 | 21 | 2008 |
| 3 | 4+3 | 71 | 6.4 | 22 | 2009 |
| 4 | 3+4 | 59 | 3.8 | 25 | 2009 |
| 5 | 4+3 | 70 | 10 | 26 | 2009 |
| 6 | 3+4 | 66 | 7.8 | 31 | 2009 |
| 7 | 4+3 | 59 | 8.5 | 49 | 2009 |
| 8 | 3+3 | 63 | 4.6 | 53 | 2009 |
| 9 | 4+5 | 54 | 32.9 | 55 | 2009 |
| 10 | 3+3 | 67 | 9.4 | 46 | 2009 |
| 11 | 4+3 | 46 | 4.5 | 57 | 2009 |
| 12 | 4+3 | 59 | 6.9 | 79 | 2009 |
| 13 | 4+3 | 55 | 4.6 | 83 | 2009 |
| 14 | 4+3 | 57 | 23 | 87 | 2009 |
| 15 | 3+3 | 69 | 4.7 | 92 | 2009 |
| 16 | 4+5 | 68 | 6.8 | 100 | 2009 |
| 31 | 4+4 | 63 | 30.4 | 289 | 2011 |
| 32 | 5+4 | 65 | 12 | 291 | 2011 |
| 33 | 4+3 | 59 | 10.5 | 296 | 2011 |
| 34 | 5+5 | 60 | 12.1 | 299 | 2011 |
| 35 | 3+4 | 61 | 9.2 | 317 | 2011 |
| 36 | 3+4 | 67 | 6.5 | 319 | 2012 |
| 37 | 4+3 | 68 | 10.1 | 320 | 2012 |
| 38 | 4+5 | 63 | 5.4 | 339 | 2012 |

**Supplementary Table 3 |** mRINs and PINs with their associated *P* values of all prostate tissues, sorted by mRIN.

| Patient ID | Tissue type |  | mRNA | |  | Protein | |
| --- | --- | --- | --- | --- | --- | --- | --- |
|  |  |  | mRIN | *P* value |  | PIN | *P* value |
| 33 | Tumor |  | -0.210 | 1.6E-40 |  | 0.955 | 0.542 |
| 35 | Tumor |  | -0.203 | 3.9E-38 |  | 0.956 | 0.649 |
| 33 | Tumor |  | -0.161 | 4.6E-25 |  | 0.949 | 0.187 |
| 35 | Benign |  | -0.127 | 7.9E-17 |  | 0.947 | 0.133 |
| 35 | Tumor |  | -0.113 | 6.2E-14 |  | 0.951 | 0.303 |
| 37 | Tumor |  | -0.096 | 1.2E-10 |  | 0.951 | 0.276 |
| 14 | Tumor |  | -0.087 | 2.6E-09 |  | 0.951 | 0.286 |
| 38 | Tumor |  | -0.073 | 3.6E-07 |  | 0.963 | 0.997 |
| 37 | Tumor |  | -0.070 | 7.9E-07 |  | 0.947 | 0.125 |
| 33 | Benign |  | -0.064 | 4.7E-06 |  | 0.951 | 0.255 |
| 38 | Benign |  | -0.063 | 6.2E-06 |  | 0.957 | 0.697 |
| 7 | Tumor |  | -0.053 | 8.6E-05 |  | 0.948 | 0.139 |
| 31 | Tumor |  | -0.043 | 8.5E-04 |  | 0.961 | 0.962 |
| 37 | Benign |  | -0.028 | 0.012 |  | 0.941 | 0.003 |
| 38 | Tumor |  | -0.026 | 0.017 |  | 0.959 | 0.901 |
| 3 | Tumor |  | -0.025 | 0.020 |  | 0.956 | 0.642 |
| 3 | Tumor |  | -0.022 | 0.028 |  | 0.949 | 0.206 |
| 7 | Benign |  | -0.019 | 0.045 |  | 0.955 | 0.588 |
| 1 | Benign |  | -0.019 | 0.045 |  | 0.953 | 0.398 |
| 15 | Tumor |  | -0.017 | 0.057 |  | 0.956 | 0.678 |
| 13 | Tumor |  | -0.014 | 0.080 |  | 0.956 | 0.629 |
| 3 | Benign |  | -0.013 | 0.087 |  | 0.942 | 0.010 |
| 6 | Tumor |  | -0.013 | 0.089 |  | 0.955 | 0.562 |
| 16 | Benign |  | -0.004 | 0.212 |  | 0.937 | 0.000 |
| 34 | Benign |  | -0.004 | 0.217 |  | 0.952 | 0.327 |
| 36 | Tumor |  | -0.003 | 0.234 |  | 0.952 | 0.316 |
| 9 | Benign |  | -0.001 | 0.273 |  | 0.952 | 0.335 |
| 34 | Tumor |  | 0.000 | 0.294 |  | 0.953 | 0.446 |
| 9 | Tumor |  | 0.001 | 0.305 |  | 0.946 | 0.101 |
| 10 | Benign |  | 0.003 | 0.347 |  | 0.954 | 0.475 |
| 6 | Benign |  | 0.004 | 0.374 |  | 0.956 | 0.686 |
| 5 | Tumor |  | 0.004 | 0.378 |  | 0.949 | 0.192 |
| 13 | Tumor |  | 0.005 | 0.406 |  | 0.953 | 0.414 |
| 31 | Tumor |  | 0.006 | 0.412 |  | 0.953 | 0.434 |
| 12 | Benign |  | 0.006 | 0.421 |  | 0.948 | 0.141 |
| 2 | Benign |  | 0.007 | 0.452 |  | 0.955 | 0.609 |
| 2 | Tumor |  | 0.008 | 0.471 |  | 0.954 | 0.513 |
| 13 | Benign |  | 0.010 | 0.512 |  | 0.957 | 0.741 |
| 15 | Benign |  | 0.011 | 0.543 |  | 0.960 | 0.932 |
| 5 | Tumor |  | 0.012 | 0.554 |  | 0.957 | 0.709 |
| 12 | Tumor |  | 0.013 | 0.576 |  | 0.941 | 0.005 |
| 36 | Tumor |  | 0.013 | 0.578 |  | 0.943 | 0.054 |
| 31 | Benign |  | 0.013 | 0.597 |  | 0.952 | 0.367 |
| 32 | Benign |  | 0.014 | 0.612 |  | 0.959 | 0.899 |
| 5 | Benign |  | 0.016 | 0.647 |  | 0.951 | 0.258 |
| 36 | Benign |  | 0.017 | 0.680 |  | 0.957 | 0.759 |
| 32 | Tumor |  | 0.017 | 0.680 |  | 0.948 | 0.166 |
| 16 | Tumor |  | 0.018 | 0.692 |  | 0.947 | 0.122 |
| 2 | Tumor |  | 0.019 | 0.715 |  | 0.951 | 0.293 |
| 4 | Benign |  | 0.019 | 0.719 |  | 0.953 | 0.382 |
| 32 | Tumor |  | 0.019 | 0.727 |  | 0.954 | 0.452 |
| 4 | Tumor |  | 0.021 | 0.761 |  | 0.956 | 0.618 |
| 1 | Tumor |  | 0.022 | 0.779 |  | 0.957 | 0.741 |
| 8 | Benign |  | 0.022 | 0.784 |  | 0.962 | 0.990 |
| 9 | Tumor |  | 0.023 | 0.798 |  | 0.951 | 0.256 |
| 11 | Benign |  | 0.024 | 0.812 |  | 0.950 | 0.234 |
| 34 | Tumor |  | 0.028 | 0.868 |  | 0.953 | 0.445 |
| 10 | Tumor |  | 0.030 | 0.895 |  | 0.940 | 0.002 |
| 16 | Tumor |  | 0.031 | 0.907 |  | 0.947 | 0.129 |
| 4 | Tumor |  | 0.031 | 0.907 |  | 0.949 | 0.192 |
| 6 | Tumor |  | 0.032 | 0.913 |  | 0.954 | 0.515 |
| 11 | Tumor |  | 0.032 | 0.915 |  | 0.958 | 0.840 |
| 12 | Tumor |  | 0.034 | 0.928 |  | 0.964 | 0.998 |
| 14 | Benign |  | 0.035 | 0.938 |  | 0.957 | 0.722 |
| 11 | Tumor |  | 0.037 | 0.953 |  | 0.957 | 0.713 |
| 14 | Tumor |  | 0.037 | 0.955 |  | 0.954 | 0.505 |
| 8 | Tumor |  | 0.039 | 0.961 |  | 0.957 | 0.722 |
| 7 | Tumor |  | 0.043 | 0.979 |  | 0.955 | 0.591 |

**Supplementary Table 4 |** Summary information of studies subjected to PIN scoring.

| **Study No.** | **Sample type** | **# MS injections** | **MS instrument** | **Acquisition method** | **Quantification** | **Storage Type** | **# total samples** | **# degraded samples** | **Raw data** |
| --- | --- | --- | --- | --- | --- | --- | --- | --- | --- |
| 1 | Breast cancer | 900 | Orbitrap Q Exactive | DDA | iTRAQ 4x | FF | 108 | 24 | Mertins P., *et al*. *Nature* 2016 [2] |
| 2 | Gastric cancer | 40 | TripleTOF 5600 | DIA | Label-free | FF | 18 | 3 | Wu J., *et al.* [3] |
| 3 | Prostate cancer | 224 | TripleTOF 5600 | DIA | Label-free | FF and FFPE | 96 | 6 | Yi Z., *et al.* [4] |
| 4 | Blood plasma | 246 | TripleTOF 5600 | DIA | Label-free | FF | 232 | 0 | Liu Y., *et al.* *Molecular Systems Biology* 2015 [5] |
| 5 | Prostate cancer | 136 | TripleTOF 5600 | DIA | Label-free | FF | 68 | 4 | Main text |

**Supplementary References**

1. Zimmerman, L.J., Li, M., Yarbrough, W.G., Slebos, R.J. & Liebler, D.C. Global stability of plasma proteomes for mass spectrometry-based analyses. *Mol Cell Proteomics* **11**, M111 014340 (2012).

2. Mertins, P., *et al.* Proteogenomics connects somatic mutations to signalling in breast cancer. *Nature* **534**, 55 (2016).

3. Wu, J., *et al.* Unpublished.

4. Zhu, Y., *et al.* Unpublished.

5. Liu, Y., *et al.* Quantitative variability of 342 plasma proteins in a human twin population. *Mol Syst Biol* **11**, 786 (2015).

6. Wang, H., Tang, H.Y., Tan, G.C. & Speicher, D.W. Data analysis strategy for maximizing high-confidence protein identifications in complex proteomes such as human tumor secretomes and human serum. *Journal of proteome research* **10**, 4993-5005 (2011).
