# Supplementary material for: Comparative analysis of mRNA and protein degradation in prostate tissues indicates high stability of proteins
Source: Nat Commun. 2019 Jun 7;10:2524. doi: 10.1038/s41467-019-10513-5 (PMC6555818; doi:10.1038/s41467-019-10513-5)
Supplement: Supplementary file 3 — Reporting Summary [file 41467_2019_10513_MOESM3_ESM.pdf]

nature research

Corresponding author(s): aebersold@imsb.biol.ethz.ch

Last updated by author(s): Apr 17, 2019

Reporting Summary

Nature Research wishes to improve the reproducibility of the work that we publish. This form provides structure for consistency and transparency in reporting. For further information on Nature Research policies, see Authors & Referees and the Editorial Policy Checklist.

Statistics

For all statistical analyses, confirm that the following items are present in the figure legend, table legend, main text, or Methods section.

n/a	Confirmed	
☐	☒	The exact sample size (n) for each experimental group/condition, given as a discrete number and unit of measurement
☐	☒	A statement on whether measurements were taken from distinct samples or whether the same sample was measured repeatedly
☐	☒	The statistical test(s) used AND whether they are one- or two-sided<br>*Only common tests should be described solely by name; describe more complex techniques in the Methods section.*
☒	☐	A description of all covariates tested
☒	☐	A description of any assumptions or corrections, such as tests of normality and adjustment for multiple comparisons
☐	☒	A full description of the statistical parameters including central tendency (e.g. means) or other basic estimates (e.g. regression coefficient) AND variation (e.g. standard deviation) or associated estimates of uncertainty (e.g. confidence intervals)
☐	☒	For null hypothesis testing, the test statistic (e.g. F, t, r) with confidence intervals, effect sizes, degrees of freedom and P value noted<br>*Give P values as exact values whenever suitable.*
☒	☐	For Bayesian analysis, information on the choice of priors and Markov chain Monte Carlo settings
☒	☐	For hierarchical and complex designs, identification of the appropriate level for tests and full reporting of outcomes
☐	☒	Estimates of effect sizes (e.g. Cohen's d, Pearson's r), indicating how they were calculated

Our web collection on statistics for biologists contains articles on many of the points above.

Software and code

Policy information about availability of computer code

Data collection	no software was used for data collection.
Data analysis	STAR aligner (v2.5) and FeatureCounts (v1.5) were used for RNA-seq data analysis. mRIN (v1.2.0) was used to estimate the degree of mRNA degradation. OpenSWATH (v2.1) was used for targeted extraction of SWATH data. PIN (v1.1) was developed to estimate the degree of proteome degradation, and was publicly available on GitHub.

For manuscripts utilizing custom algorithms or software that are central to the research but not yet described in published literature, software must be made available to editors/reviewers. We strongly encourage code deposition in a community repository (e.g. GitHub). See the Nature Research guidelines for submitting code & software for further information.

Data

Policy information about availability of data

All manuscripts must include a data availability statement. This statement should provide the following information, where applicable:

- Accession codes, unique identifiers, or web links for publicly available datasets
- A list of figures that have associated raw data
- A description of any restrictions on data availability

RNA-Seq data have been deposited in the Sequence Read Archive (BioProject number: PRJNA414084). Regarding proteomic analysis of the clinical cohort, the SWATH raw data and analyzed data as well as assay libraries are deposited to the ProteomeXchange Consortium via the PRIDE partner repository with the data set identifier PXD007841. Regarding the benchmarking study, the SWATH raw data and analyzed data as well as assay libraries are deposited to the ProteomeXchange Consortium via the PRIDE partner repository with the data set identifier PXD013622.

Field-specific reporting

Please select the one below that is the best fit for your research. If you are not sure, read the appropriate sections before making your selection.

☒ Life sciences ☐ Behavioural & social sciences ☐ Ecological, evolutionary & environmental sciences

For a reference copy of the document with all sections, see nature.com/documents/nr-reporting-summary-flat.pdf

Life sciences study design

All studies must disclose on these points even when the disclosure is negative.

Sample size	From the prostate cancer ProCOC cohort, we selected 24 patients with complete clinical information and high quality samples collected from different age groups, Gleason groups and PSA-relapse-based survival. The sample size was determined as 24 to achieve sufficient statistical power for transcriptomics and proteomics study of molecular degradation.
Data exclusions	No data was excluded.
Replication	Samples for proteomic study were analyzed with replicates, and achieved high degree of reproducibility.
Randomization	Tissue samples were randomly allocated for the measurements according to the age group, Gleason score and PSA-relapse-based survival.
Blinding	The investigators were blinded to group allocation during data collection and analysis.

Reporting for specific materials, systems and methods

We require information from authors about some types of materials, experimental systems and methods used in many studies. Here, indicate whether each material, system or method listed is relevant to your study. If you are not sure if a list item applies to your research, read the appropriate section before selecting a response.

Materials & experimental systems

n/a	Involved in the study	
☒	☐	Antibodies
☐	☒	Eukaryotic cell lines
☒	☐	Palaeontology
☒	☐	Animals and other organisms
☒	☐	Human research participants
☒	☐	Clinical data

Methods

n/a	Involved in the study	
☒	☐	ChIP-seq
☒	☐	Flow cytometry
☒	☐	MRI-based neuroimaging

Eukaryotic cell lines

Policy information about cell lines

Cell line source(s)	HeLa cells at Ruedi Aebersold lab were purchased from ATCC.
Authentication	The cell authentication was performed by SNV profiles.
Mycoplasma contamination	All the cells before any proteomic studies were confirmed to be mycoplasma negative.
Commonly misidentified lines (See ICLAC register)	No commonly misidentified cell lines were used.
